# Machine-Learning-Based Prediction of Cervical Pedicle Screw Malposition from Clinical and Anatomical Features

**DOI:** 10.3390/jcm15155972

**Published:** 2026-07-31

**Authors:** Milan S. Vosko, Stefan Aspalter, Anja Blenk, Petra Böhm, Nico Stroh-Holly, Andreas Gruber, Wolfgang Senker

**Affiliations:** 1 Department of Neurosurgery, Kepler University Hospital, Johannes Kepler University Linz, 4040 Linz, Austria; milan.vosko2@kepleruniklinikum.at (M.S.V.); asblenk@icloud.com (A.B.); petra.boehm@kepleruniklinikum.at (P.B.); andreas.gruber_1@kepleruniklinikum.at (A.G.); wolfgang.senker@kepleruniklinikum.at (W.S.); 2 Clinical Research Institute for Neuroscience, Johannes Kepler University Linz, 4040 Linz, Austria

**Keywords:** cervical pedicle screws, outcome prediction, machine learning, minimally invasive spine surgery

## Abstract

**Background/Objectives**: Cervical pedicle screw (CPS) placement provides superior biomechanical stability but remains technically demanding and associated with a risk of screw malposition. While recent advances in imaging and navigation have improved placement accuracy, reliable prediction of malposition remains challenging. The aim of this study was to evaluate whether machine learning (ML) models can predict CPS malposition using structured clinical and anatomical features. **Methods**: We performed a retrospective analysis of 862 pedicle screws from 168 posterior cervical spine surgeries conducted at our institution between 2018 and 2025. Clinical, procedural, and anatomical variables, including age, sex, body size parameters, surgical indication, vertebral level, pedicle angle, and pedicle width, were evaluated. Pedicle morphology was partially derived from CT-based automated segmentation using TotalSegmentator (v2.13.0), while selected anatomical parameters were manually measured. Supervised ML models, including Random Forest, Balanced Random Forest, XGBoost (v3.2.0), Support Vector Machine, and K-Nearest Neighbor, were trained and compared using Python and scikit-learn to predict inaccurate screw placement. Model performance was evaluated using Area Under the Receiver Operating Characteristic Curve (ROC AUC), F1-score, precision, and recall. Model interpretability was assessed using Shapley Additive Explanations (SHAP). **Results**: The dataset showed a clinically representative class distribution, with 91.1% of screws classified as acceptable and 8.9% as inaccurate. Across all models, predictive performance was moderate and consistent. Balanced Random Forest achieved the highest discriminative performance (ROC AUC 0.69) and provided the most balanced classification profile, while other models demonstrated comparable overall performance with varying sensitivity to the minority class. SHAP analysis identified anatomical and procedural variables, including pedicle width and angle, as relevant contributors to model output. Feature contributions were distributed across variables, with substantial overlap between outcome groups. **Conclusions**: ML-based prediction of CPS malposition using clinical and anatomical features demonstrates consistent and interpretable performance. The results highlight that predictive performance is primarily influenced by dataset characteristics, including class distribution and feature overlap, rather than model selection alone. This study provides an important baseline for ML-based CPS prediction and supports future research integrating larger datasets and more detailed anatomical representations to enhance predictive accuracy.

## 1. Introduction

Cervical pedicle screw (CPS) fixation provides superior biomechanical stability compared to alternative posterior fixation techniques such as lateral mass screws and is therefore widely used in the treatment of cervical spine instability [1,2]. However, CPS placement remains technically demanding due to complex cervical spine anatomy, the small pedicle diameter and its close proximity to critical neurovascular structures, resulting in a non-negligible risk of screw malposition and associated complications [1,2,3,4,5,6].

Reported perforation rates for CPS placement vary widely. While navigation- and robot-assisted systems have improved accuracy, they have not completely eliminated the risk of misplacement. Consequently, accurate identification of risk factors for screw malposition remains of high clinical relevance [5,7,8,9,10].

While recent advances in image-based modeling have enabled detailed morphometric analyses, most studies focus on descriptive or planning tasks rather than predictive modeling of screw malposition [6,11,12].

It remains unclear whether structured clinical variables and CT morphometric anatomical parameters, such as pedicle angles and pedicle width, are sufficient to enable meaningful prediction of CPS malposition using machine learning approaches.

To date, no study has systematically evaluated the predictive performance of multiple machine learning models using exclusively clinical and radiological features for this purpose.

Therefore, the aim of this study was to develop a predictive model using ML algorithms to objectively anticipate cervical pedicle screw malposition based on clinical data and pedicle-related parameters.

By addressing this question, this study aims not only to assess the feasibility of ML-based prediction in this context but also to provide insight into the limitations of current feature representations for modeling CPS accuracy.

## 2. Materials and Methods

### 2.1. Ethics Committee Approval

The retrospective single-center study was conducted at the Department of Neurosurgery, Kepler University Hospital, Linz, Austria, and the ethical approval was obtained from JKU Faculty of Medicine’s Ethics Committee of the Federal State of Upper Austria: EK Nr: 1024/2025 (approval date: 9 April 2025).

### 2.2. Patient Population and Data Collection

This retrospective study utilized a dataset consisting of 862 pedicle screws from 168 posterior cervical spine surgeries performed at our institution between 2018 and 2025. Preoperative variables were derived from the medical records of each patient. Screw position was graded using the Gertzbein–Robbins classification [13] on intraoperative O-arm-CT imaging with the following distribution:

For the purpose of predictive modeling, outcomes were binarized into acceptable screw placement (grades 0–1) and inaccurate screw placement (grades 2–3), consistent with common clinical practice. Minor cortical breaches (<2 mm) were therefore considered acceptable, while larger deviations were considered clinically relevant due to the increased risk of neurovascular injury.

The dataset used in the present study corresponds to the cervical pedicle screw cohort previously described by Aspalter et al. (J. Clin. Med. 2025, 14, 6597) [14]. While the underlying patient data are identical, the scientific objective of the present work is entirely distinct, focusing on machine-learning-based prediction of screw malposition rather than descriptive accuracy reporting.

All patient data were fully anonymized, and the study was approved by the institutional ethics review board.

### 2.3. Features and Data Processing

We systematically gathered a broad range of features to form the basis of the dataset, including demographic variables such as sex, body height and Body Mass Index (BMI). Regarding the surgical details we focused on the reference array required for the navigation system, the vertebral level of screw insertion, the sequence of screw implantation, the number of pedicle screws placed during surgery, the surgical indication, the pedicle angle and pedicle width.

To estimate pedicle morphology, an automated segmentation approach was applied to the preoperative CT datasets using TotalSegmentator [15], an open-source deep learning framework for anatomical structure segmentation in cross-sectional imaging developed by Wasserthal et al.

Vertebral segmentations generated by TotalSegmentator were processed using a custom Python feature extraction pipeline. Because fully automated pedicle localization proved insufficiently robust during preliminary development, pedicle locations were manually identified using anatomical landmark points. These landmarks served only to initialize the automated computation of pedicle width measurements from the vertebral segmentation masks. Prior to feature extraction, segmentation masks underwent automated quality-control procedures to identify fragmented or anatomically implausible segmentations.

Missing numerical values were imputed using median imputation, while categorical variables were imputed using the most frequent category. Numerical variables were standardized prior to model training, and categorical variables were encoded using one-hot encoding.

### 2.4. Statistical Analysis

#### Predictive Modeling

The primary outcome was pedicle screw placement accuracy according to the Gertzbein–Robbins classification, dichotomized into acceptable (grades 0–1) and inaccurate (grades 2–3) placement. We evaluated multiple supervised ML classifiers, including tree-based models (Random Forest and XGBoost), an ensemble approach (Balanced Random Forest), and distance- and kernel-based methods (K-Nearest Neighbor and Support Vector Machine), aiming to predict inaccurate CPS placement during surgery.

Prior to the final benchmarking analysis, hyperparameters for each classifier were optimized using a cross-validated grid search performed during a preliminary model development stage. The optimal hyperparameter configuration identified for each model was subsequently fixed and used throughout the comparative evaluation. All preprocessing steps were incorporated within a machine learning pipeline to prevent information leakage. Within each training fold, missing numerical values were imputed using the median and categorical variables using the most frequent category. Numerical variables were standardized, while categorical variables were transformed using one-hot encoding.

Given the pronounced class imbalance, the Synthetic Minority Oversampling Technique (SMOTE) [16] was applied exclusively to the training data within each cross-validation fold for all models except Balanced Random Forest, which inherently accounts for class imbalance through balanced bootstrap sampling.

Model interpretability was assessed using SHAP [17], allowing evaluation of the contribution of individual features to model predictions. Model performance was evaluated using ten-fold stratified group cross-validation. Grouping ensured that all pedicle screws originating from the same patient were assigned to the same fold, thereby preventing patient-level information leakage between training and testing data. Model performance was assessed using accuracy, precision, recall, F1-score, and ROC AUC. ROC AUC was used as a threshold-independent measure of discrimination, reflecting the ability of each model to distinguish between accurate and inaccurate screw placement across different decision thresholds. Given the pronounced class imbalance, particular emphasis was placed on the F1-score, which provides a balanced measure of predictive performance for the minority class.

All analyses were conducted using Python 3.11 (Python Software Foundation, Wilmington, DE, USA).

ML model training also with scikit-learn v1.8.0 (Inria, Paris, France) to predict inaccurate screw placement.

## 3. Results

A total of 862 pedicle screws from posterior cervical spine surgeries were included in the analysis. Screw placement accuracy was classified according to the Gertzbein–Robbins classification, with the majority (91.1%) of screws categorized as acceptable (grades 0–1) and a substantially smaller proportion (only 8.9%) classified as inaccurate (grades 2–3), resulting in a pronounced class imbalance.

**Figure 1:**
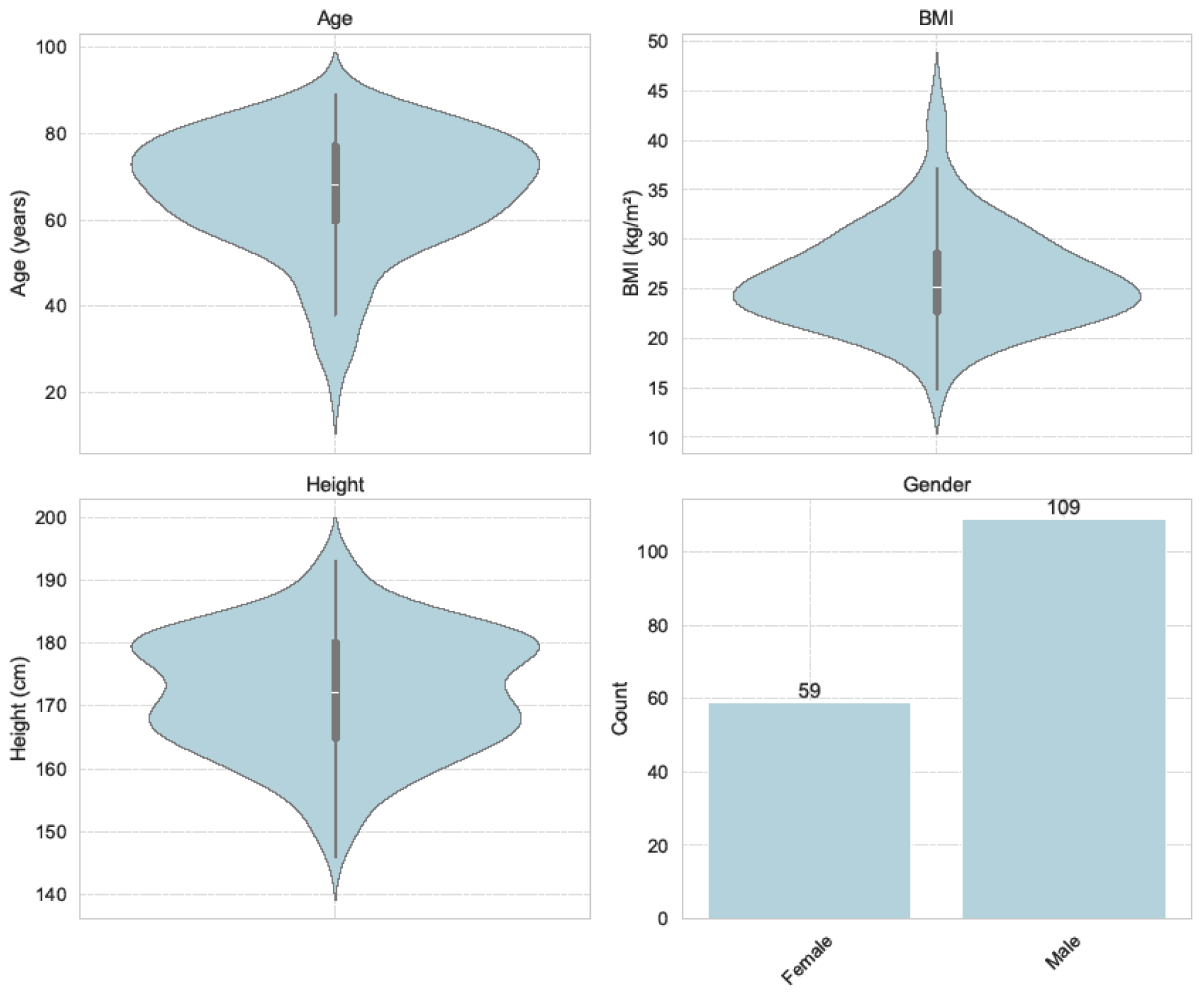
Distribution of cohort characteristics including age, BMI, body height, and sex. Continuous variables are shown as violin plots with median and interquartile range, and categorical variables as counts.

The mean patient age 66.7 [57.5–75.9] years, with a predominance of male patients (109 male vs. 59 female). The average BMI was 26.0 [22.6–29.4] kg/m^2^ and the mean body height was 172.2 [165.8–178.6] cm (Figure 1). The median number of pedicle screws per surgery was 8.0 [4.0–10.0].

The most frequent surgical indication was trauma (44.6%) followed by degenerative spinal disease (24.4%) and neoplastic conditions (9.5%), with other indications being less commonly represented. Pedicle screws were most commonly placed at subaxial cervical levels, particularly at C4–C6, with decreasing frequency toward the upper cervical and thoracic segments. The reference array was predominantly positioned at C2 (55.4%) and, to a lesser extent, at C6 (17.3%), reflecting standard intraoperative workflows.

### 3.1. Prediction of Screw Malposition—Machine Learning

Across all evaluated machine learning models, predictive performance remained moderate but consistent, with only limited separation between accurate and inaccurate screw placement. The Balanced Random Forest model demonstrated the highest overall discriminative performance, achieving a ROC AUC of 0.69, followed by Random Forest (0.65) and XGBoost (0.65). K-nearest neighbors and support vector machine models achieved ROC AUC values of 0.63 and 0.62, respectively.

While Random Forest and XGBoost showed high overall accuracy (>0.89), both models demonstrated limited sensitivity for detecting inaccurate screws, reflected by low recall values (0.04 and 0.08, respectively). In contrast, the Support Vector Machine achieved the highest recall (0.59), indicating improved detection of the minority class, although this occurred at the expense of substantially lower specificity and overall accuracy. The K-nearest neighbors model similarly demonstrated increased sensitivity (recall 0.35) with comparatively lower overall discrimination.

Balanced Random Forest provided the most balanced performance profile, combining the highest ROC AUC (0.69) with improved minority-class detection (recall 0.27) and a higher F1-score (0.18) compared with conventional Random Forest and XGBoost models. Overall, these findings suggest that cervical pedicle screw malposition can be partially predicted using demographic, procedural, and anatomical variables, although substantial overlap between outcome groups limits discriminative performance.

**Figure 2:**
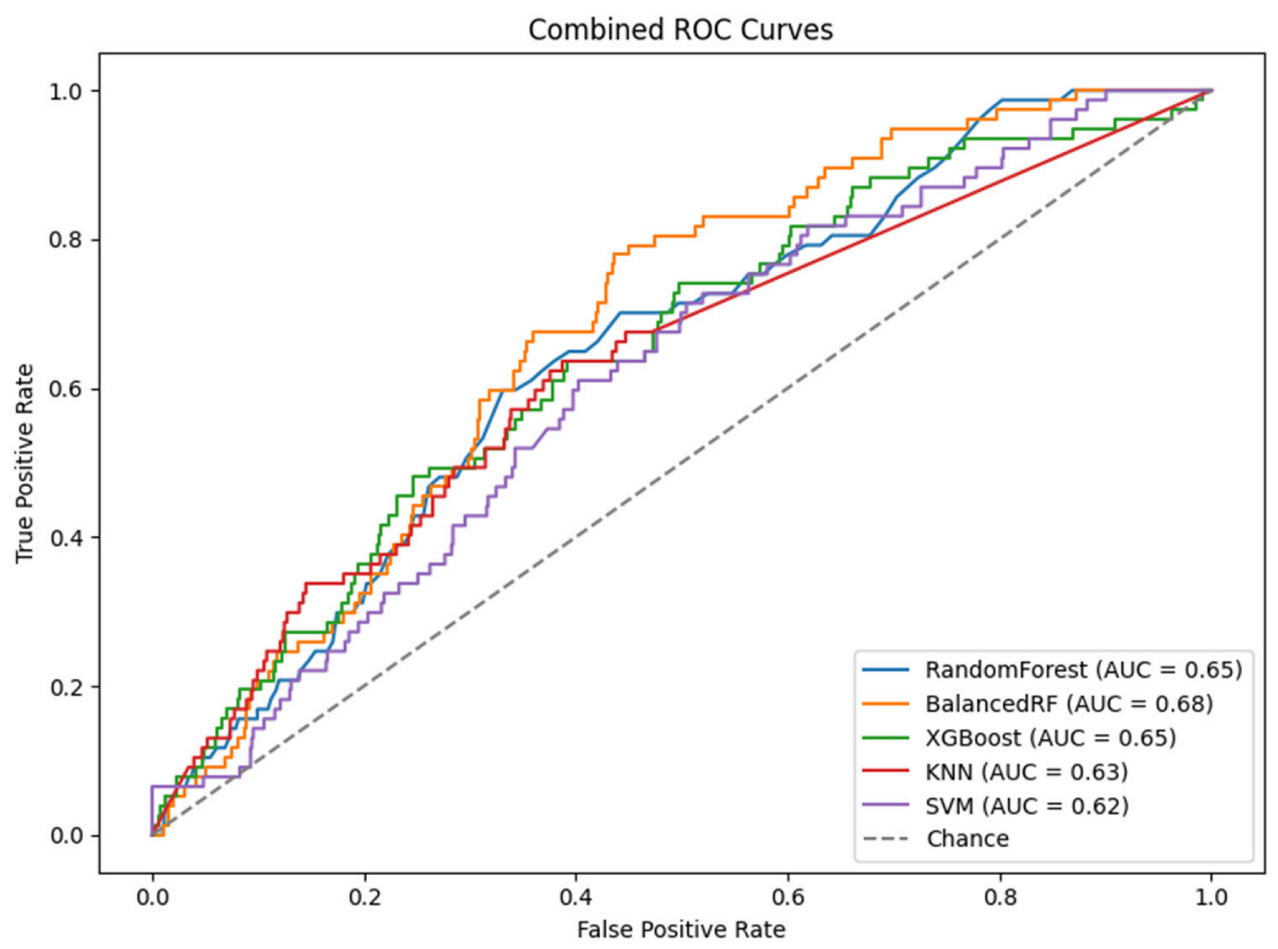
ROC curves of the evaluated ML models for prediction of CPS malposition. The curves illustrate the relationship between the true positive rate (sensitivity) and the false positive rate (1—specificity) across different classification thresholds. All evaluated models, including Random Forest, Balanced Random Forest, XGBoost, K-nearest neighbors, and Support Vector Machine, are shown. The dashed diagonal line represents random classification (no discrimination). ROC AUC reflects the overall discriminative performance of each model.

The combined ROC curves (Figure 2) demonstrated that all models operated within a relatively narrow performance range, with only moderate deviation from the reference line. This pattern suggests considerable overlap in the underlying feature distributions between accurate and inaccurate screw placement. Detailed performance metrics are summarized in Table 1.

### 3.2. Feature Importance and SHAP Analysis

Feature contribution analysis using SHAP demonstrated that both anatomical and procedural variables contributed to model predictions in a distributed manner across classifiers. In the Balanced Random Forest model, the most influential variables included screw order, vertebral level (particularly T1), pedicle screw count, pedicle angle, pedicle width measurements, BMI, and body height. Certain clinical variables, such as neoplastic disease indication and reference array location at C6, also contributed to model output.

Pedicle morphology features, including median pedicle width and estimated minimum pedicle width, consistently appeared among the most influential anatomical predictors, supporting the relevance of pedicle anatomy for prediction of screw placement accuracy. However, SHAP value distributions demonstrated substantial overlap between correctly and incorrectly classified screws across all features, and no single variable consistently dominated model predictions. Overall SHAP value magnitudes remained relatively small and comparable across variables, indicating that predictive information was distributed across multiple informative features rather than driven by a single dominant predictor.

**Figure 3:**
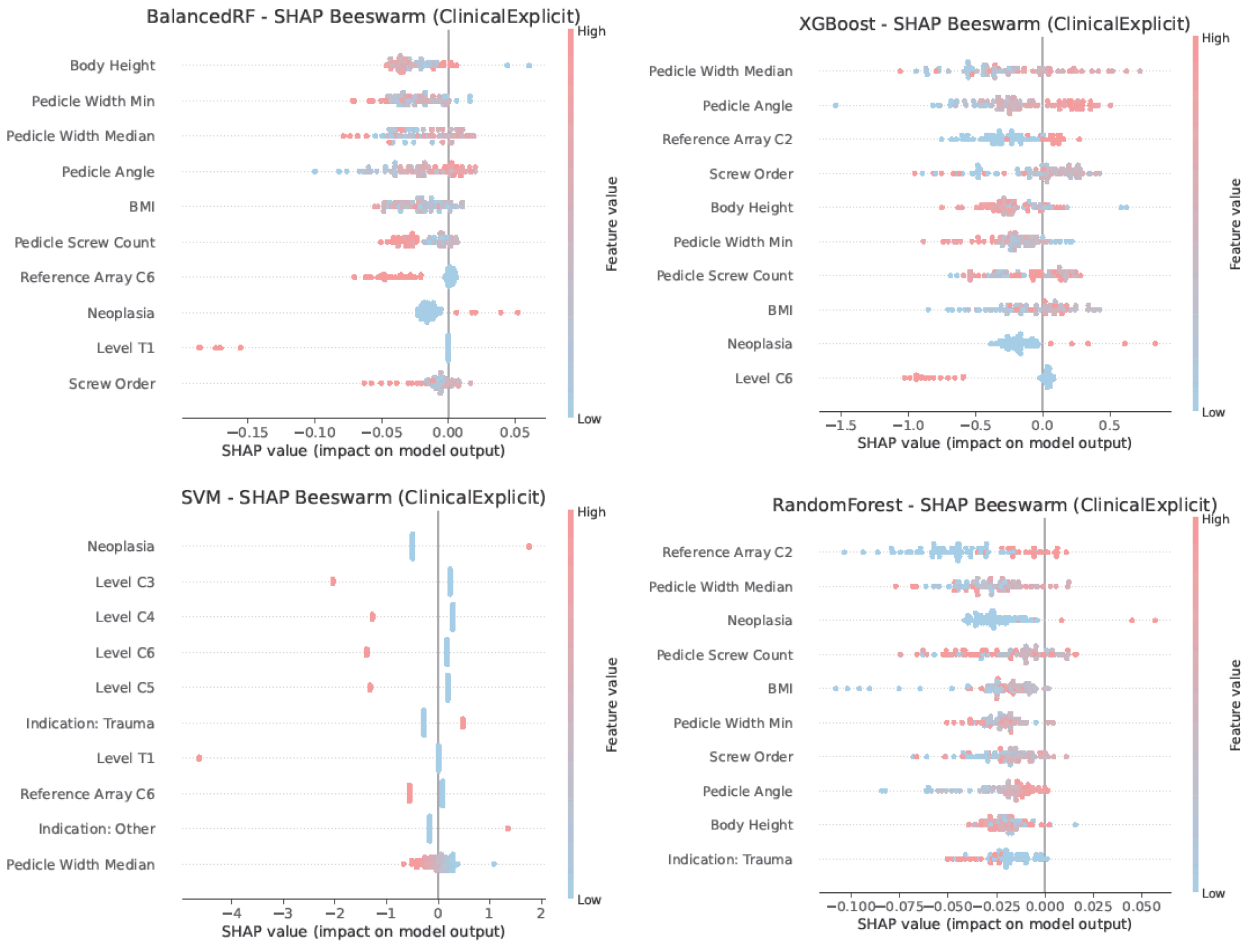
SHAP summary (beeswarm) plots illustrating feature contributions across the evaluated machine learning models (Random Forest, Balanced Random Forest, XGBoost, and Support Vector Machine). Each point represents an individual screw, with color indicating feature value and position reflecting the impact on model output.

Overall, similar feature contribution patterns were observed across classifiers, suggesting a stable and reproducible feature importance structure despite only moderate predictive performance. SHAP summary plots for all evaluated models are presented in Figure 3.

## 4. Discussion

### 4.1. Principal Findings

In this study, we evaluated the performance of multiple machine learning models for predicting CPS malposition using structured clinical and anatomical features. Across all evaluated models, predictive performance was observed within a relatively narrow and comparable range, with moderate discrimination but considerable variability in sensitivity across classifiers.

A central finding of this analysis is that model performance was consistent across different algorithmic approaches, without a clear advantage of any individual method. This pattern suggests that predictive capacity in this setting is primarily influenced by the structure and separability of the available data, rather than by model selection itself. From a methodological perspective, this represents an important insight, indicating that model performance in CPS prediction from our dataset is inherently linked to the characteristics of the feature space.

### 4.2. Interpretation of Findings

The observed performance characteristics can be interpreted in the context of the underlying data structure. While several models achieved moderate ROC AUC values, this did not consistently translate into improved identification of inaccurately placed screws. This reflects a common pattern in imbalanced datasets, where discrimination and sensitivity may vary depending on threshold selection and class distribution.

Importantly, the similarity of performance across models suggests that predictive information contained in the available features is distributed rather than concentrated, and that no single feature or subset of features provides a dominant signal for classification. This observation is further supported by SHAP analysis, which demonstrated consistent but diffuse feature contributions and overlapping value distributions across outcome groups.

### 4.3. Relation to the Existing Literature

Previous studies have consistently highlighted the importance of pedicle-specific anatomical characteristics for safe screw placement. Fukushima et al. demonstrated that reduced pedicle width is strongly associated with increased perforation rates, alongside patient-related factors and surgical experience.

Similarly, earlier biomechanical studies have established that CPS placement depends on precise alignment within a narrow and highly variable pedicle corridor [1,2,3]. Inaccurate screw trajectory or insufficient pedicle dimensions have been identified as primary causes of cortical breach and neurovascular injury [1].

More recent studies emphasize the complexity of cervical pedicle morphology, including variability in pedicle shape, cortical thickness and angular orientation [6,18,19].

Recent machine learning applications in spine surgery are often relying on high-resolution CT data and deep learning frameworks [11,12,20]. These models incorporate direct representations of anatomical structures, which were not fully captured in the present dataset. In addition, pedicle morphology parameters derived from automated segmentation may be subject to variability in precision compared to manually measured features.

### 4.4. Clinical Implications

From a clinical perspective, the findings indicate that the predictive behavior observed in this study reflects, to a large extent, the underlying distribution of the outcome variable. The relatively low proportion of inaccurately placed screws results in a limited number of positive cases, which constrains the ability of machine learning models to learn stable and generalizable patterns of misplacement.

In this context, the variability of model performance across classifiers and thresholds can be understood as a consequence of limited representation of the minority class. This is consistent with the observation that no model demonstrated consistent superiority, and that sensitivity remained dependent on trade-offs with specificity.

At the same time, the analysis suggests that clinically relevant variables, including pedicle-related parameters and procedural factors, contribute in a distributed and context-dependent manner, without forming clearly separable groups within the available feature space. This reflects the multifactorial nature of CPS placement, where outcome is influenced by interacting anatomical, procedural, and technical factors.

Existing studies have shown that intraoperative guidance techniques, such as navigation or O-arm-based systems, improve screw placement accuracy but do not eliminate misplacement entirely [7,18,19,21]. This suggests that even under optimized technical conditions, CPS placement remains influenced by anatomical and individual variability that may not be fully captured in simplified representations.

### 4.5. Imaging-Derived Features and Segmentation

Imaging-derived parameters were obtained using different approaches. Pedicle width was extracted based on automated segmentation using TotalSegmentator, while pedicle angles were measured manually on CT images. Additional morphometric parameters, such as pedicle diameter or cortical thickness, could not be consistently used due to variability in segmentation accuracy. As a result, only selected imaging-derived variables were included in the final analysis.

## 5. Limitations

This study has several limitations. The retrospective, monocentric design may limit generalizability, and external validation was not performed but should be addressed in future studies. The pronounced class imbalance reflects real-world data but introduces challenges for model training and evaluation.

Although anatomical measurements were computed automatically, pedicle localization required manual landmark placement. Consequently, some observer dependence remains. The reproducibility of landmark placement was not formally evaluated and should be investigated in future studies.

Furthermore, additional anatomically relevant variables, such as pedicle cortical thickness, pedicle length and volume, or vertebral body measurements, were not included and may provide additional information in future analyses.

Finally, the limited number of inaccurately placed screws represents a key constraint in the present dataset, as it restricts the ability of machine learning models to learn stable and generalizable patterns.

Future work would benefit from the development of multicentric datasets with a larger number of misplacement cases and standardized clinical and anatomical variables. Such datasets could improve both predictive modeling and the understanding of factors associated with accurate screw placement.

## 6. Conclusions

This study provides a systematic and, to our knowledge, is one of the first comprehensive evaluations of ML-based prediction of CPS malposition using structured clinical and anatomical data. The results demonstrate that predictive performance in this setting is strongly influenced by the underlying data structure, particularly the limited availability of misplacement cases.

Rather than indicating a limitation of ML methods alone, these findings highlight fundamental aspects of predictive modeling in surgical contexts, where rare adverse events and complex anatomical interactions shape model behavior. The moderate discriminative performance and low F1-scores observed indicate that, at this stage, ML-based malposition prediction using clinically accessible features does not yet reach the threshold for direct clinical application. These findings should therefore be interpreted as an evidence-based baseline, defining the current boundaries of prediction in this setting rather than proposing a ready-to-use clinical tool.

By identifying these constraints, this study lays the groundwork for future research integrating larger, multicentric datasets and more detailed anatomical representations. Such developments may enable more robust predictive models and contribute to improved planning and precision in CPS.

## Figures and Tables

**Table 1:** Performance metrics of the evaluated machine learning models for prediction of cervical pedicle screw malposition. Presented values include accuracy, precision, recall, F1-score, and ROC AUC. Accuracy reflects the overall proportion of correctly classified screws, while precision indicates the proportion of correctly predicted inaccurate screws among all positive predictions. Recall represents the sensitivity of the models in detecting inaccurately placed screws. The F1-score combines precision and recall, providing a balanced measure of model performance in the presence of class imbalance. ROC AUC indicates the overall discriminative ability of each model across different classification thresholds.

Model	Accuracy	Precision	Recall	F1	ROC AUC
Random Forest	0.90	0.12	0.04	0.06	0.65
Balanced Random Forest	0.79	0.14	0.27	0.18	0.69
XG Boost	0.90	0.28	0.08	0.11	0.65
KNN	0.76	0.15	0.35	0.21	0.63
SVM	0.60	0.13	0.59	0.21	0.62

## Data Availability

The data that support the findings of this study are available from the corresponding author, upon reasonable request.

## Abbreviations

The following abbreviations are used in this manuscript:

AI	Artificial Intelligence
BMI	Body Mass Index
BRF	Balanced Random Forest
CNN	Convolutional Neural Network
CPS	Cervical Pedicle Screw
CT	Computed Tomography
CV	Cross-Validation
F1	F1-score
HU	Hounsfield Units
KNN	K-Nearest Neighbors
ML	Machine Learning
RF	Random Forest
ROC	Receiver Operating Characteristic
ROC AUC	Area Under the Receiver Operating Characteristic Curve
SHAP	Shapley Additive Explanations
SMOTE	Synthetic Minority Over-sampling Technique
SVM	Support Vector Machine
